# Supplementary material for: Deep phenotyping of Alzheimer’s disease leveraging electronic medical records identifies sex-specific clinical associations
Source: Nat Commun. 2022 Feb 3;13:675. doi: 10.1038/s41467-022-28273-0 (PMC8814236; doi:10.1038/s41467-022-28273-0)
Supplement: Supplementary file 1 — Supplementary Information [file 41467_2022_28273_MOESM1_ESM.pdf]

Deep Phenotyping of Alzheimer's Disease Leveraging Electronic Medical Records Identifies Sex-Specific Clinical Associations

Supplementary Information

Alice S. Tang[1,2,3]*, Tomiko Oskotsky[1,4], Shreyas Havaldar[5], William G. Mantyh[6], Mesude Bicak[5,7], Caroline Warly Solsberg[8,9,10], Sarah Woldemariam[1], Billy Zeng[3], Zicheng Hu[1], Boris Oskotsky[1], Dena Dubal[9], Isabel E. Allen[10], Benjamin S. Glicksberg[5,7], Marina Sirota[1,4]*

Supplementary Tables

	Count	Age	Death Status	Race	
**Alzheimer's Cohort**	**6,612**	**86.4<br>90 (83-91)**	**Alive: 6714 (76.3%)<br>Deceased: 2090 (23.7%)**	**White/Caucasian: 5462 (64.0%)<br>Asian: 879 (10.3%)<br>Black/African American: 586 (6.9%)<br>Hawaiian/Pacific Islander: 452 (5.3%)**	**American Native: 9 (.1%)<br>Other: 743 (8.7%)<br>Unknown/Declined: 802 (4.7%)**
Males	2,382 (36.0%)	85.7<br>90 (84-91)	Alive: 1778 (74.6%)<br>Deceased: 604 (25.4%)	White/Caucasian: 1570 (66.8%)<br>Asian: 254 (10.8%)<br>Black/African American: 128 (5.4%)<br>Hawaiian/Pacific Islander: 117 (5.0%)	Other: 209 (8.9%)<br>Unknown/Declined: 72 (3.1%)
Females	4,223 (63.9%)	86.8<br>90 (82-91)	Alive: 3084 (73%)<br>Deceased: 1139 (27%)	White/Caucasian: 2525 (60.5%)<br>Asian: 497 (11.9%)<br>Black/African American: 393 (9.4%)<br>Hawaiian/Pacific Islander: 217 (5.2%)	American Native: 8 (0.2%)<br>Other: 404 (9.7%)<br>Unknown/Declined: 130 (3.1%)
Other or Unknown	7 (0.10%)	90.7<br>91 (90.5-91)	Alive: 7 (100%)	White/Caucasian: 6 (85.7%)<br>Unknown/Declined: 1 (14.3%)	
**Control Cohort**	**13,224**	**86.2<br>90 (83 – 91)**	**Alive: 13432 (76.3%)<br>Deceased: 4176 (23.7%)**	**White/Caucasian: 10924 (64.0%)<br>Asian: 1759 (10.3%)<br>Black/African American: 1172 (6.9%)<br>Hawaiian/Pacific Islander: 904 (5.3%)**	**American Native: 18 (.1%)<br>Other: 1487 (8.7%)<br>Unknown/Declined: 802 (4.7%)**
Males	4,674 (35.3%)	85.8<br>90 (82-91)	Alive: 3248 (69.5%)<br>Deceased: 1426 (30.5%)	White/Caucasian: 3076 (66.8%)<br>Asian: 490 (10.7%)<br>Black/African American: 277 (6.0%)<br>Hawaiian/Pacific Islander: 222 (4.8%)	American Native: 5 (.1%)<br>Other: 384 (8.3%)<br>Unknown/Declined: 247 (3.2%)
Females	8,539 (64.6%)	86.5<br>90 (84-91)	Alive: 6253 (73.2%)<br>Deceased: 2286 (26.8%)	White/Caucasian: 5225 (61.9%)<br>Asian: 1024 (12.1%)<br>Black/African American: 768 (9.1%)<br>Hawaiian/Pacific Islander: 387 (4.6%)	American Native: 11 (.1%)<br>Other: 783 (9.3%)<br>Unknown/Declined: 246 (2.9%)
Other or Unknown	11 (0.10%)	90.2<br>90 (90-91)	Alive: 10 (90.9%)<br>Deceased: 1 (9.1%)	White/Caucasian: 1 (11.1%)<br>Other: 3 (33.3%)	Unknown/Declined: 5 (55.6%)

Supplementary Table 1. Patient Demographics with Encounter Thresholds and Controlling.

Distribution of sex, estimated age, death status, and first race among Alzheimer's and control cohorts. These cohorts are thresholded on more than 10 encounters, and over a year representation in the EMR. Patients are matched at a 1:2 Alzheimer to control ratio with the demographics shown in the table. Estimated age shows mean and median (25%ile - 75%ile).

AD (Alzheimer's disease) (HCC)
Alzheimer disease (HCC)
Alzheimer disease type 3 (HCC)
Alzheimer's dementia (HCC)
Alzheimer's dementia with behavioral disturbance (HCC)
Alzheimer's dementia with behavioral disturbance, unspecified timing of dementia onset (HCC)
Alzheimer's dementia without behavioral disturbance (HCC)
Alzheimer's dementia without behavioral disturbance, unspecified timing of dementia onset (HCC)
Alzheimer's dementia, late onset (HCC)
Alzheimer's dementia, late onset, with behavioral disturbance (HCC)
Alzheimer's disease (HCC)
Alzheimer's disease of other onset
Alzheimer's disease of other onset with behavioral disturbance (HCC)
Alzheimer's disease of other onset without behavioral disturbance (HCC)
Alzheimer's disease with delirium (HCC)
Alzheimer's disease with early onset (CODE) (HCC)
Alzheimer's disease with early onset (HCC)
Alzheimer's disease with late onset (CODE) (HCC)
Alzheimer's disease with late onset (HCC)
Alzheimer's disease with presenile onset (HCC)
Alzheimer's disease, early onset (HCC)
Alzheimer's disease, familial (HCC)
Alzheimer's disease, focal onset (HCC)
Alzheimer's disease, unspecified (CODE) (HCC)
Alzheimer's disease, unspecified (HCC)
Alzheimer's type dementia (HCC)
Alzheimer's type dementia with late onset with behavioral disturbance (HCC)
Alzheimer's type dementia with late onset without behavioral disturbance (HCC)
Alzheimers disease (HCC)
DAT (dementia Alzheimer type)
DAT (dementia of Alzheimer type) (HCC)
Dementia due to Alzheimer's disease (HCC)
Dementia in Alzheimer's disease (HCC)
Dementia in Alzheimer's disease with delusions (HCC)
Dementia in Alzheimer's disease with depression (HCC)
Dementia in Alzheimer's disease with early onset (HCC)
Dementia in Alzheimer's disease with early onset with behavioral disturbance (HCC)
Dementia in Alzheimer's disease with early onset without behavioral disturbance (HCC)
Dementia in Alzheimer's disease with early onset, with behavioral disturbance
Dementia in Alzheimer's disease with early onset, without behavioral disturbance
Dementia in Alzheimer's disease with late onset
Dementia of Alzheimer's type with behavioral disturbance (HCC)
Dementia of Alzheimer's type, with early onset, with depressed mood (HCC)
Dementia of the Alzheimer's type (HCC)
Dementia of the Alzheimer's type with early onset with behavioral disturbance (HCC)
Dementia of the Alzheimer's type with late onset without behavioral disturbance (HCC)
Dementia of the Alzheimer's type without behavioral disturbance (HCC)
Dementia of the Alzheimer's type, with late onset, uncomplicated (HCC)
Dementia of the Alzheimer's type, with late onset, with delirium (HCC)
Dementia of the Alzheimer's type, with late onset, with delusions (HCC)
Dementia of the Alzheimer's type, with late onset, with depressed mood (HCC)
Dementia of the Alzheimer's type, with late onset, with depressive mood (HCC)
Dementia, Alzheimer's, with behavior disturbance (HCC)
Early onset Alzheimer disease
Early onset Alzheimer's dementia without behavioral disturbance (HCC)
Early onset Alzheimer's disease with behavioral disturbance (HCC)
Family history of Alzheimer's disease
Focal Alzheimer's disease (HCC) 'Late onset Alzheimer disease (HCC)
Late onset Alzheimer's disease with behavioral disturbance (HCC)
Late onset Alzheimer's disease without behavioral disturbance (HCC)
Major neurocognitive disorder due to Alzheimer's disease (HCC)
Major neurocognitive disorder due to Alzheimer's disease, possible (HCC)
Major neurocognitive disorder due to Alzheimer's disease, probable, with behavioral disturbance (HCC)
Major neurocognitive disorder due to Alzheimer's disease, probable, without behavioral disturbance (HCC)
Major neurocognitive disorder due to Alzheimer's disease, with behavioral disturbance (HCC)
Major neurocognitive disorder due to possible Alzheimer's disease (HCC)
Major neurocognitive disorder, due to Alzheimer's disease, with behavioral disturbance, mild (HCC)
Major neurocognitive disorder, due to Alzheimer's disease, without behavioral disturbance, mild (HCC)
Major neurocognitive disorder, due to Alzheimer's disease, without behavioral disturbance, moderate (HCC)
Major neurocognitive disorder, due to Alzheimer's disease, without behavioral disturbance, severe (HCC)
Mild major neurocognitive disorder due to Alzheimer's disease with behavioral disturbance (HCC)
Mild major neurocognitive disorder due to Alzheimer's disease without behavioral disturbance (HCC)
Mild neurocognitive disorder due to Alzheimer's disease (HCC)
Mild possible major neurocognitive disorder due to Alzheimer's disease (HCC)
Mixed Alzheimer's and vascular dementia (HCC)
Mixed Alzheimer's and vascular dementia with behavior disturbances (HCC)
Moderate major neurocognitive disorder due to Alzheimer's disease without behavioral disturbance (HCC)
Moderate probable major neurocognitive disorder due to Alzheimer's disease with behavioral disturbance
Other Alzheimer's disease (HCC)
Possible major neurocognitive disorder due to Alzheimer's disease
Primary degenerative dementia of Alzheimer type (HCC)
Primary degenerative dementia of the Alzheimer type, senile onset (HCC)
Primary degenerative dementia of the Alzheimer type, senile onset, uncomplicated (HCC)
Primary degenerative dementia of the Alzheimer type, senile onset, with depression (HCC)
Probable major neurocognitive disorder due to Alzheimer's disease with behavioral disturbance
Probable major neurocognitive disorder due to Alzheimer's disease without behavioral disturbance
Progressive aphasia in Alzheimer's disease (HCC)
SDAT (senile dementia of Alzheimer's type) (HCC)
Senile dementia of Alzheimer's type (HCC)
Sporadic Alzheimer's disease (HCC)'

Supplementary Table 2. UMAP Exclusion Terms

Table of diagnosis excluded in UMAP embedding. These terms contain the word ‘Alzheimer’.

UCSF: Graph (>1%)	Number Nodes	Number Edges	Avg Number Neighbors	Network Diameter	Network Radius	Characteristic Path Length	Clustering Coefficient	Network density	Network heterogeneity	Network Centralization	Connected Components	Singletons
ADDiagnosisNameAll	1056	27504	62.15	4	2	2.043	0.830	0.070	1.626	0.763	171	169
ADDiagnosisNameFemale	962	25459	61.64	4	2	2.038	0.832	0.075	1.586	0.761	137	136
ADDiagnosisNameMale	924	20102	52.90	4	2	2.057	0.823	0.070	1.633	0.739	164	162
ADL3NameALL	483	23505	97.33	2	1	1.798	0.899	0.202	1.054	0.801	1	0
ADL3NameFemale	452	21958	97.16	2	1	1.785	0.899	0.215	1.021	0.788	1	0
ADL3NameMale	445	20099	90.33	2	1	1.797	0.899	0.203	1.046	0.800	1	0
ADL2NameALL	165	7960	96.48	2	1	1.412	0.896	0.588	0.479	0.417	1	0
ADL2NameFemale	160	7531	94.73	2	1	1.400	0.897	0.600	0.469	0.406	2	1
ADL2NameMale	158	7257	91.86	2	1	1.415	0.892	0.585	0.481	0.420	1	0
ConDiagnosisNameAll	421	2445	18.04	4	2	2.048	0.738	0.067	1.671	0.843	151	150
ConDiagnosisNameFemale	167	2109	25.72	3	2	1.892	0.848	0.158	1.156	0.797	4	3
ConDiagnosisNameMale	321	1417	13.43	4	2	2.078	0.681	0.064	1.717	0.815	111	110
ConL3NameALL	318	5772	43.89	2	1	1.832	0.760	0.168	1.135	0.839	56	55
ConL3NameFemale	190	5434	57.20	2	1	1.697	0.830	0.303	0.829	0.705	1	0
ConL3NameMale	282	4195	37.46	3	2	1.837	0.750	0.168	1.125	0.835	59	58
ConL2NameALL	150	3990	55.80	2	1	1.607	0.854	0.393	0.697	0.616	8	7
ConL2NameFemale	122	3760	61.64	2	1	1.491	0.866	0.509	0.552	0.499	1	0
ConL2NameMale	137	3200	48.48	2	1	1.630	0.862	0.370	0.726	0.640	6	5

Mount Sinai: Graph (>1%)	Number Nodes	Number Edges	Avg Number Neighbors	Network Diameter	Network Radius	Characteristic Path Length	Clustering Coefficient	Network density	Network heterogeneity	Network Centralization	Connected Components	Singletons
ADDiagnosisNameAll	483	1788	15.96	4	2	2.030	0.782	0.072	1.696	0.756	260	259
ADDiagnosisNameFemale	482	1753	15.72	4	2	2.035	0.769	0.071	1.700	0.751	260	259
ADDiagnosisNameMale	446	1034	12.16	4	2	2.084	0.722	0.072	1.674	0.700	277	276
ADL3NameALL	348	10434	59.97	2	1	1.827	0.910	0.173	1.152	0.832	1	0
ADL3NameFemale	352	10145	59.68	2	1	1.824	0.909	0.176	1.142	0.829	13	12
ADL3NameMale	332	8162	52.32	2	1	1.832	0.905	0.168	1.166	0.837	21	20
ADL2NameALL	141	4625	65.60	2	1	1.531	0.875	0.469	0.608	0.539	1	0
ADL2NameFemale	141	4480	64.93	2	1	1.526	0.876	0.474	0.602	0.534	4	3
ADL2NameMale	139	4037	59.37	2	1	1.560	0.878	0.440	0.642	0.569	4	3
ConDiagnosisNameAll	461	13	3.25	3	2	1.607	0.558	0.464	0.527	0.524	454	453
ConDiagnosisNameFemale	461	13	3.25	3	2	1.607	0.558	0.464	0.527	0.524	454	453
ConDiagnosisNameMale	423	13	3.25	3	2	1.607	0.558	0.464	0.527	0.524	416	415
ConL3NameALL	347	1038	17.16	2	1	1.857	0.788	0.143	1.267	0.871	227	226
ConL3NameFemale	351	1038	17.16	2	1	1.857	0.788	0.143	1.267	0.871	231	230
ConL3NameMale	331	980	16.47	3	2	1.863	0.780	0.140	1.280	0.867	213	212
ConL2NameALL	141	1323	32.67	3	2	1.594	0.877	0.408	0.677	0.568	61	60
ConL2NameFemale	141	1323	32.67	3	2	1.594	0.877	0.408	0.677	0.568	61	60
ConL2NameMale	139	1290	31.85	3	2	1.606	0.865	0.398	0.690	0.566	59	58

UCSF Comparison	Nodes	Mann Whitney U Test	Avg Shortest Path Length	Betweeness Centrality	Closeness Centrality	Clustering Coefficient	Degree	Eccentricity	Neighborhood Connectivity	Number Undirected Edges	Stress Centrality	Topological Coefficient
Alzheimer vs Control: Diagnosis Name	All Nodes	Stat	2.68E+05	2.46E+05	2.63E+05	2.73E+05	3.07E+05	3.22E+05	3.73E+05	3.07E+05	2.56E+05	2.81E+05
		pval	3.75E-10	9.15E-05	2.31E-08	1.07E-12	1.02E-30	6.87E-48	1.58E-93	1.02E-30	2.48E-08	6.20E-16
		Δmean	3.96E-01	-1.52E-03	9.88E-02	2.20E-01	4.05E+01	1.01E+00	2.57E+02	4.05E+01	1.17E+04	1.35E-01
	Singletons Removed	Stat	1.23E+05	8.88E+03	1.18E+05	8.99E+04	1.61E+05	1.76E+05	2.28E+05	1.61E+05	1.89E+04	9.85E+04
		pval	6.05E-01	4.64E-11	6.05E-01	7.51E-01	1.22E-17	3.06E-42	4.30E-110	1.22E-17	1.31E-02	5.76E-02
		Δmean	-7.26E-03	-9.16E-03	2.03E-03	1.81E-02	4.40E+01	4.97E-01	2.76E+02	4.40E+01	3.19E+04	3.63E-02
Alzheimer vs Control: L3 Name	All Nodes	Stat	8.30E+04	8.19E+04	9.71E+04	1.02E+05	1.10E+05	9.02E+04	1.39E+05	1.10E+05	8.70E+04	1.13E+05
		pval	5.19E-02	7.44E-02	2.16E-10	3.00E-17	2.15E-25	6.25E-21	2.69E-85	2.15E-25	3.50E-04	4.21E-29
		Δmean	2.83E-01	-9.79E-04	1.08E-01	2.70E-01	6.10E+01	3.47E-01	1.76E+02	6.10E+01	6.20E+03	2.50E-01
	Singletons Removed	Stat	5.65E+04	1.03E+04	7.06E+04	5.77E+04	8.36E+04	6.36E+04	1.13E+05	8.36E+04	1.55E+04	6.79E+04
		pval	0.0121	0.0274	1.21E-02	1.03E-01	9.42E-13	6.64E-01	4.02E-69	9.42E-13	4.96E-05	2.53E-08
		Δmean	-3.44E-02	-3.70E-03	1.27E-02	1.80E-02	5.34E+01	1.73E-03	1.49E+02	5.34E+01	1.31E+04	1.00E-01
Alzheimer vs Control: L2 Name	All Nodes	Stat	8.40E+03	1.39E+04	1.75E+04	1.26E+04	1.88E+04	1.29E+04	2.06E+04	1.88E+04	1.50E+04	1.72E+04
		pval	8.40E-07	5.70E-02	2.06E-10	7.73E-01	1.51E-15	3.52E-02	3.65E-24	1.51E-15	9.45E-04	2.48E-09
		Δmean	-1.20E-01	-1.58E-03	1.23E-01	8.24E-02	4.33E+01	8.79E-02	3.31E+01	4.33E+01	7.29E+02	1.39E-01
	Singletons Removed	Stat	7.24E+03	3.93E+03	1.64E+04	1.08E+04	1.77E+04	1.17E+04	1.94E+04	1.77E+04	5.03E+03	1.54E+04
		pval	5.14E-09	0.11	5.14E-09	0.362	5.46E-14	6.51E-01	1.64E-22	5.46E-14	2.07E-01	3.12E-07
		Δmean	-1.95E-01	-4.17E-03	9.26E-02	1.80E-02	4.07E+01	-5.13E-03	2.85E+01	4.07E+01	4.30E+02	8.91E-02
Female AD vs Male AD: Diagnosis Name	All Nodes	Stat	4.57E+05	4.69E+05	4.88E+05	4.73E+05	4.85E+05	3.41E+05	5.22E+05	4.85E+05	4.72E+05	4.78E+05
		pval	5.15E-01	7.16E-01	6.35E-02	4.96E-01	9.92E-02	3.39E-31	2.96E-06	9.92E-02	4.90E-01	2.93E-01
		Δmean	1.98E-02	-8.89E-05	1.02E-02	1.69E-02	8.26E+00	-2.87E-01	4.19E+01	8.26E+00	2.75E+03	1.39E-02
	Singletons Removed	Stat	3.09E+05	5.33E+04	3.39E+05	2.68E+05	3.37E+05	1.92E+05	3.74E+05	3.37E+05	5.67E+04	2.73E+05
		pval	1.01E-01	4.56E-01	1.01E-01	9.79E-01	1.71E-01	1.10E-73	9.98E-08	1.71E-01	5.32E-01	5.68E-01
		Δmean	-1.50E-02	-3.69E-04	2.79E-03	1.27E-03	8.82E+00	-4.06E-01	4.32E+01	8.82E+00	6.85E+03	6.75E-03
Female AD vs Male AD: L3 Name	All Nodes	Stat	1.05E+05	1.04E+05	1.04E+05	1.05E+05	1.07E+05	1.04E+05	1.13E+05	1.07E+05	1.05E+05	1.06E+05
		pval	9.12E-01	9.13E-01	9.12E-01	9.61E-01	5.28E-01	9.74E-01	3.08E-02	5.28E-01	9.59E-01	7.47E-01
		Δmean	2.59E-03	-7.91E-05	-7.28E-04	-8.94E-04	3.28E+00	1.05E-04	1.36E+01	3.28E+00	9.47E+02	-2.65E-04
	Singletons Removed	Stat	1.05E+05	2.01E+04	1.04E+05	1.03E+05	1.07E+05	1.04E+05	1.13E+05	1.07E+05	2.07E+04	1.04E+05
		pval	0.912	0.461	9.12E-01	9.70E-01	5.28E-01	9.74E-01	3.08E-02	5.28E-01	8.03E-01	7.53E-01
		Δmean	2.59E-03	-2.17E-04	-7.28E-04	-1.29E-03	3.28E+00	1.05E-04	1.36E+01	3.28E+00	1.95E+03	-5.36E-04
Female AD vs Male AD: L2 Name	All Nodes	Stat	1.24E+04	1.27E+04	1.29E+04	1.27E+04	1.31E+04	1.26E+04	1.36E+04	1.31E+04	1.28E+04	1.29E+04
		pval	7.59E-01	9.08E-01	7.59E-01	9.74E-01	5.62E-01	9.92E-01	2.61E-01	5.62E-01	8.70E-01	7.35E-01
		Δmean	-7.12E-03	-7.87E-05	3.60E-03	5.89E-03	2.30E+00	1.58E-04	2.10E+00	2.30E+00	-6.22E+01	3.46E-03
	Singletons Removed	Stat	1.24E+04	5.31E+03	1.29E+04	1.27E+04	1.31E+04	1.26E+04	1.36E+04	1.31E+04	5.35E+03	1.29E+04
		pval	7.59E-01	0.429	7.59E-01	0.974	5.62E-01	0.992	2.61E-01	5.62E-01	0.48	7.35E-01
		Δmean	-7.12E-03	-3.26E-04	3.60E-03	5.89E-03	2.30E+00	1.58E-04	2.10E+00	2.30E+00	-2.47E+02	3.46E-03
Female: AD vs Control: Diagnosis Name	All Nodes	Stat	2.47E+05	2.23E+05	2.34E+05	2.47E+05	2.78E+05	2.49E+05	3.40E+05	2.78E+05	2.33E+05	2.55E+05
		pval	3.15E-11	1.16E-04	1.98E-06	6.28E-12	8.80E-29	3.89E-18	5.56E-91	8.80E-29	4.00E-08	3.53E-15
		Δmean	3.93E-01	-1.55E-03	9.58E-02	2.18E-01	3.99E+01	6.46E-01	2.45E+02	3.99E+01	1.14E+04	1.36E-01
	Singletons Removed	Stat	1.17E+05	8.17E+03	1.04E+05	8.21E+04	1.47E+05	1.19E+05	2.10E+05	1.47E+05	1.74E+04	9.03E+04
		pval	1.53E-01	1.32E-10	1.53E-01	7.09E-01	1.68E-16	2.64E-04	1.15E-107	1.68E-16	1.43E-02	5.98E-02
		Δmean	-1.64E-03	-9.37E-03	1.03E-04	1.76E-02	4.33E+01	8.01E-02	2.62E+02	4.33E+01	3.09E+04	3.67E-02
Female: AD vs Control: L3 Name	All Nodes	Stat	7.79E+04	7.70E+04	9.01E+04	9.57E+04	1.02E+05	8.41E+04	1.30E+05	1.02E+05	8.16E+04	1.05E+05
		pval	5.75E-02	7.26E-02	3.23E-09	3.11E-16	3.67E-23	1.91E-19	1.25E-80	3.67E-23	4.57E-04	2.62E-27
		Δmean	2.72E-01	-1.00E-03	1.03E-01	2.67E-01	5.77E+01	3.32E-01	1.67E+02	5.77E+01	5.77E+03	2.46E-01
	Singletons Removed	Stat	5.40E+04	9.75E+03	6.63E+04	5.42E+04	7.85E+04	6.02E+04	1.06E+05	7.85E+04	1.44E+04	6.36E+04
		pval	0.023	0.019	2.30E-02	1.23E-01	1.05E-11	6.68E-01	1.26E-65	1.05E-11	3.64E-04	9.73E-08
		Δmean	-3.09E-02	-3.80E-03	1.15E-02	1.81E-02	5.04E+01	1.75E-03	1.42E+02	5.04E+01	1.19E+04	9.75E-02
Female: AD vs Control: L2 Name	All Nodes	Stat	7.93E+03	1.29E+04	1.64E+04	1.22E+04	1.76E+04	1.21E+04	1.92E+04	1.76E+04	1.39E+04	1.64E+04
		pval	1.25E-06	1.10E-01	1.13E-09	5.29E-01	1.39E-14	6.37E-02	3.75E-22	1.39E-14	2.95E-03	8.95E-10
		Δmean	-1.25E-01	-1.58E-03	1.18E-01	8.53E-02	4.07E+01	7.65E-02	3.05E+01	4.07E+01	6.27E+02	1.42E-01
	Singletons Removed	Stat	6.97E+03	3.97E+03	1.54E+04	1.04E+04	1.67E+04	1.11E+04	1.82E+04	1.67E+04	5.00E+03	1.47E+04
		pval	1.70E-08	0.253	1.70E-08	0.573	2.92E-13	0.645	9.58E-21	2.92E-13	0.106	1.27E-07
		Δmean	-1.91E-01	-3.84E-03	9.11E-02	1.91E-02	3.84E+01	-5.36E-03	2.65E+01	3.84E+01	4.35E+02	9.01E-02
Male: AD vs Control: Diagnosis Name	All Nodes	Stat	1.76E+05	1.67E+05	1.81E+05	1.91E+05	2.11E+05	2.16E+05	2.52E+05	2.11E+05	1.74E+05	1.94E+05
		pval	1.84E-05	1.18E-03	2.82E-07	5.34E-13	3.46E-26	3.29E-34	1.28E-71	3.46E-26	9.63E-07	2.09E-14
		Δmean	3.44E-01	-2.21E-03	8.95E-02	2.38E-01	3.47E+01	9.30E-01	2.19E+02	3.47E+01	9.11E+03	1.42E-01
	Singletons Removed	Stat	7.94E+04	6.72E+03	8.42E+04	5.83E+04	1.15E+05	1.19E+05	1.56E+05	1.15E+05	1.43E+04	6.18E+04
		pval	5.14E-01	5.87E-07	5.14E-01	6.58E-01	2.12E-19	8.54E-33	8.81E-91	2.12E-19	4.09E-05	1.02E-01
		Δmean	-2.25E-02	-1.24E-02	5.42E-03	2.49E-02	3.92E+01	4.96E-01	2.43E+02	3.92E+01	2.60E+04	3.46E-02
Male: AD vs Control: L3 Name	All Nodes	Stat	6.90E+04	6.68E+04	8.31E+04	8.75E+04	9.24E+04	5.06E+04	1.18E+05	9.24E+04	7.20E+04	9.50E+04
		pval	3.34E-02	1.31E-01	5.52E-13	1.91E-20	4.43E-26	1.11E-09	8.58E-88	4.43E-26	3.40E-04	9.71E-31
		Δmean	3.35E-01	-1.19E-03	1.28E-01	3.03E-01	6.11E+01	4.82E-03	1.77E+02	6.11E+01	5.27E+03	2.74E-01
	Singletons Removed	Stat	4.31E+04	8.47E+03	5.72E+04	4.67E+04	6.65E+04	2.47E+04	9.23E+04	6.65E+04	1.36E+04	5.42E+04
		pval	0.00297	0.0189	2.97E-03	2.26E-02	5.72E-12	6.79E-61	1.56E-70	5.72E-12	1.35E-06	1.52E-08
		Δmean	-4.15E-02	-4.27E-03	1.46E-02	2.35E-02	5.34E+01	-5.09E-01	1.50E+02	5.34E+01	1.12E+04	1.11E-01
Male: AD vs Control: L2 Name	All Nodes	Stat	6.83E+03	1.17E+04	1.56E+04	1.11E+04	1.69E+04	1.12E+04	1.88E+04	1.69E+04	1.29E+04	1.49E+04
		pval	4.73E-08	2.10E-01	6.05E-11	7.50E-01	1.41E-16	9.67E-02	1.55E-27	1.41E-16	3.31E-03	2.52E-08
		Δmean	-1.56E-01	-2.01E-03	1.24E-01	6.13E-02	4.51E+01	6.76E-02	3.51E+01	4.51E+01	8.48E+02	1.29E-01
	Singleton Removed	Stat	6.04E+03	3.88E+03	1.48E+04	9.94E+03	1.61E+04	1.04E+04	1.80E+04	1.61E+04	5.08E+03	1.38E+04
		pval	6.97E-10	0.808	6.97E-10	0.635	2.09E-15	0.674	2.50E-26	2.09E-15	0.00134	5.65E-07
		Δmean	-2.15E-01	-4.23E-03	1.01E-01	1.66E-02	4.34E+01	-5.08E-03	3.19E+01	4.34E+01	1.02E+03	9.47E-02
Male vs Female Controls: Diagnosis Name	All Nodes	Stat	6.07E+04	6.58E+04	7.05E+04	6.87E+04	6.88E+04	6.59E+04	7.18E+04	6.88E+04	6.63E+04	6.85E+04
		pval	6.60E-02	9.93E-01	8.92E-02	2.69E-01	2.80E-01	9.75E-01	3.14E-02	2.80E-01	8.26E-01	3.22E-01
		Δmean	-2.94E-02	-7.56E-04	3.84E-03	3.70E-02	3.02E+00	-2.01E-03	1.58E+01	3.02E+00	4.12E+02	1.96E-02
	Singletons Removed	Stat	2.30E+04	3.08E+03	3.28E+04	1.83E+04	3.11E+04	2.81E+04	3.40E+04	3.11E+04	3.54E+03	1.80E+04
		pval	9.78E-04	6.06E-01	9.78E-04	6.06E-01	3.14E-02	7.59E-01	3.46E-05	3.14E-02	2.82E-02	8.31E-01
		Δmean	-3.59E-02	-3.42E-03	8.11E-03	8.57E-03	4.71E+00	1.03E-02	2.48E+01	4.71E+00	1.99E+03	4.64E-03
Male vs Female Controls: L3 Name	All Nodes	Stat	4.40E+04	4.32E+04	4.66E+04	4.59E+04	4.67E+04	3.06E+04	5.20E+04	4.67E+04	4.39E+04	4.62E+04
		pval	8.32E-01	8.45E-01	1.48E-01	2.39E-01	1.30E-01	4.27E-13	4.51E-05	1.30E-01	8.74E-01	1.97E-01
		Δmean	6.56E-02	-2.72E-04	2.37E-02	3.49E-02	6.69E+00	-3.27E-01	2.38E+01	6.69E+00	4.43E+02	2.81E-02
	Singletons Removed	Stat	2.76E+04	5.40E+03	3.02E+04	2.17E+04	3.03E+04	1.42E+04	3.56E+04	3.03E+04	6.04E+03	2.20E+04
		pval	0.403	0.645	4.03E-01	4.86E-01	3.55E-01	6.24E-39	1.06E-05	3.55E-01	3.41E-01	3.75E-01
		Δmean	-8.03E-03	-6.85E-04	2.41E-03	4.14E-03	6.26E+00	-5.11E-01	2.23E+01	6.26E+00	1.27E+03	1.31E-02
Male vs Female Controls: L2 Name	All Nodes	Stat	9.32E+03	9.95E+03	1.06E+04	9.74E+03	1.09E+04	9.96E+03	1.16E+04	1.09E+04	1.02E+04	9.95E+03
		pval	3.26E-01	9.35E-01	3.96E-01	6.97E-01	1.73E-01	8.68E-01	2.06E-02	1.73E-01	8.08E-01	9.43E-01
		Δmean	-3.71E-02	-5.06E-04	9.92E-03	-1.81E-02	6.74E+00	-8.75E-03	6.73E+00	6.74E+00	1.59E+02	-9.02E-03
	Singleton Removed	Stat	8.61E+03	3.17E+03	9.87E+03	8.76E+03	1.02E+04	9.24E+03	1.09E+04	1.02E+04	3.38E+03	8.97E+03
		pval	3.31E-01	0.761	3.31E-01	0.976	1.30E-01	0.971	1.15E-02	1.30E-01	0.291	0.758
		Δmean	-3.09E-02	-7.11E-04	1.34E-02	3.35E-03	7.26E+00	4.33E-04	7.45E+00	7.26E+00	3.42E+02	8.03E-03

Color Legend
pval < .05
pval < .01
pval < .001
pval < .0001
positive Δmean
negative Δmean

Mount Sinai Comparison	Nodes	Mann Whitney U Test	Avg Shortest Path Length	Betweeness Centrality	Closeness Centrality	Clustering Coefficient	Degree	Eccentricity	Neighborhood Connectivity	Number Undirected Edges	Stress Centrality	Topological Coefficient
Alzheimer vs Control: Diagnosis Name	All Nodes	Stat	1.62E+05	1.28E+05	1.60E+05	1.55E+05	1.61E+05	1.62E+05	1.62E+05	1.61E+05	1.29E+05	1.55E+05
		pval	6.92E-57	9.38E-17	2.49E-54	1.21E-48	1.91E-56	6.59E-58	1.48E-57	1.91E-56	3.27E-17	1.27E-47
		Δmean	9.13E-01	3.95E-04	2.20E-01	3.53E-01	7.35E+00	1.37E+00	5.33E+01	7.35E+00	4.23E+02	2.08E-01
	Singletons Removed	Stat	1.49E+03	1.20E+01	3.06E+02	7.93E+02	1.28E+03	1.47E+03	1.79E+03	1.28E+03	2.68E+02	3.96E+02
		pval	1.55E-03	1.98E-03	1.55E-03	1.04E-01	3.82E-02	3.72E-09	1.56E-06	3.82E-02	2.04E-02	1.74E-01
		Δmean	4.23E-01	-1.89E-01	-1.49E-01	1.49E-01	1.27E+01	6.79E-01	1.10E+02	1.27E+01	2.57E+03	-1.13E-01
Alzheimer vs Control: L3 Name	All Nodes	Stat	9.83E+04	7.77E+04	1.01E+05	1.02E+05	1.09E+05	9.98E+04	1.19E+05	1.09E+05	7.97E+04	1.05E+05
		pval	4.40E-48	4.50E-17	2.23E-55	6.00E-62	4.78E-78	1.82E-74	1.90E-113	4.78E-78	9.06E-21	4.25E-66
		Δmean	1.18E+00	-1.21E-04	3.65E-01	6.35E-01	5.40E+01	1.30E+00	1.95E+02	5.40E+01	3.59E+03	4.59E-01
	Singletons Removed	Stat	1.96E+04	1.87E+03	2.25E+04	1.82E+04	3.04E+04	2.11E+04	4.06E+04	3.04E+04	3.82E+03	2.10E+04
		pval	0.263	0.000503	2.63E-01	8.41E-01	4.24E-13	7.67E-01	2.67E-52	4.24E-13	2.76E-03	3.08E-02
		Δmean	-2.98E-02	-1.61E-02	1.02E-02	1.04E-02	4.28E+01	2.52E-03	1.45E+02	4.28E+01	7.72E+03	5.50E-02
Alzheimer vs Control: L2 Name	All Nodes	Stat	1.34E+04	1.20E+04	1.49E+04	1.41E+04	1.69E+04	1.35E+04	1.96E+04	1.69E+04	1.28E+04	1.49E+04
		pval	2.63E-07	6.47E-04	3.71E-13	3.72E-10	1.79E-24	3.02E-12	1.69E-45	1.79E-24	1.92E-06	4.61E-13
		Δmean	6.15E-01	-5.00E-04	3.05E-01	3.71E-01	4.68E+01	7.80E-01	7.09E+01	4.68E+01	1.43E+03	3.33E-01
	Singletons Removed	Stat	4.99E+03	1.27E+03	6.43E+03	5.63E+03	8.44E+03	5.07E+03	1.11E+04	8.44E+03	2.09E+03	6.41E+03
		pval	1.18E-01	0.15	1.18E-01	0.856	3.22E-09	0.00012	6.39E-32	3.22E-09	0.00077	0.128
		Δmean	-6.30E-02	-7.58E-03	2.93E-02	-1.83E-03	3.29E+01	-1.13E-01	4.71E+01	3.29E+01	2.31E+03	3.40E-02
Female AD vs Male AD: Diagnosis Name	All Nodes	Stat	1.14E+05	1.09E+05	1.19E+05	1.17E+05	1.18E+05	1.15E+05	1.24E+05	1.18E+05	1.09E+05	1.16E+05
		pval	8.97E-02	5.42E-01	2.08E-03	5.16E-03	4.75E-03	3.29E-02	8.66E-06	4.75E-03	4.39E-01	1.02E-02
		Δmean	1.47E-01	-2.93E-04	4.45E-02	8.06E-02	2.64E+00	2.25E-01	2.12E+01	2.64E+00	1.52E+02	4.46E-02
	Singletons Removed	Stat	1.64E+04	1.99E+03	2.15E+04	1.44E+04	2.05E+04	1.78E+04	2.65E+04	2.05E+04	2.40E+03	1.37E+04
		pval	2.31E-02	6.72E-02	2.31E-02	2.50E-01	1.52E-01	4.96E-02	1.26E-11	1.52E-01	9.25E-01	8.51E-01
		Δmean	-4.90E-02	-3.85E-03	1.03E-02	2.26E-02	3.56E+00	-6.38E-02	3.12E+01	3.56E+00	7.35E+02	3.32E-03
Female AD vs Male AD: L3 Name	All Nodes	Stat	5.94E+04	5.86E+04	6.06E+04	6.06E+04	6.26E+04	5.98E+04	6.77E+04	6.26E+04	5.93E+04	6.14E+04
		pval	7.17E-01	9.39E-01	4.12E-01	3.51E-01	1.03E-01	1.60E-01	3.56E-04	1.03E-01	7.02E-01	2.43E-01
		Δmean	4.04E-02	-1.67E-04	1.72E-02	2.79E-02	8.47E+00	4.96E-02	2.42E+01	8.47E+00	7.39E+02	2.51E-02
	Singletons Removed	Stat	5.24E+04	9.11E+03	5.36E+04	5.34E+04	5.57E+04	5.29E+04	6.07E+04	5.57E+04	9.81E+03	5.42E+04
		pval	8.06E-01	6.18E-01	8.06E-01	8.18E-01	2.63E-01	6.15E-01	1.36E-03	2.63E-01	5.75E-01	5.84E-01
		Δmean	-7.80E-03	-5.46E-04	2.77E-03	1.46E-03	7.36E+00	-2.68E-03	1.97E+01	7.36E+00	1.67E+03	6.90E-03
Female AD vs Male AD: L2 Name	All Nodes	Stat	9.16E+03	9.68E+03	1.04E+04	9.94E+03	1.06E+04	9.80E+03	1.07E+04	1.06E+04	9.79E+03	1.03E+04
		pval	0.348	0.853	0.344	0.824	0.251	0.984	0.206	0.251	0.991	0.446
		Δmean	-0.033	-0.000305	0.016	-0.00158	5.46	0.000816	3.44	5.46	1.58E+02	0.0148
	Singletons Removed	Stat	8.74E+03	2.79E+03	1.00E+04	9.52E+03	1.02E+04	9.39E+03	1.02E+04	1.02E+04	2.90E+03	9.90E+03
		pval	0.33	0.518	0.33	0.821	0.238	0.991	0.193	0.238	0.278	0.434
		Δmean	-0.0342	-0.000165	0.0161	-0.00189	5.56	0.000213	3.49	5.56	4.69E+02	0.0149
Female: AD vs Control: Diagnosis Name	All Nodes	Stat	1.61E+05	1.28E+05	1.60E+05	1.54E+05	1.61E+05	1.61E+05	1.61E+05	1.61E+05	1.28E+05	1.54E+05
		pval	1.05E-56	2.60E-16	4.01E-54	1.67E-47	3.26E-56	1.07E-57	2.35E-57	3.26E-56	9.41E-17	1.74E-46
		Δmean	9.14E-01	4.12E-04	2.19E-01	3.46E-01	7.22E+00	1.37E+00	5.27E+01	7.22E+00	4.13E+02	2.03E-01
	Singletons Removed	Stat	1.49E+03	1.20E+01	2.96E+02	7.81E+02	1.26E+03	1.46E+03	1.78E+03	1.26E+03	2.59E+02	3.80E+02
		pval	1.35E-03	2.04E-03	1.35E-03	9.68E-02	4.74E-02	3.99E-09	1.58E-06	4.74E-02	2.27E-02	1.57E-01
		Δmean	4.28E-01	-1.89E-01	-1.51E-01	1.49E-01	1.25E+01	6.79E-01	1.10E+02	1.25E+01	2.57E+03	-1.17E-01
Female: AD vs Control: L3 Name	All Nodes	Stat	9.85E+04	7.88E+04	1.02E+05	1.03E+05	1.09E+05	1.00E+05	1.19E+05	1.09E+05	8.07E+04	1.05E+05
		pval	3.74E-44	2.28E-16	5.53E-52	1.98E-57	1.26E-72	7.58E-67	7.20E-105	1.26E-72	8.06E-20	6.21E-62
		Δmean	1.12E+00	-1.28E-04	3.50E-01	6.07E-01	5.17E+01	1.24E+00	1.83E+02	5.17E+01	3.40E+03	4.42E-01
	Singletons Removed	Stat	1.89E+04	1.85E+03	2.22E+04	1.78E+04	2.97E+04	2.06E+04	3.95E+04	2.97E+04	3.73E+03	2.06E+04
		pval	0.197	0.000661	1.97E-01	8.13E-01	3.61E-13	7.82E-01	2.38E-51	3.61E-13	3.23E-03	2.55E-02
		Δmean	-3.31E-02	-1.60E-02	1.14E-02	9.48E-03	4.25E+01	2.38E-03	1.41E+02	4.25E+01	7.53E+03	5.71E-02
Female: AD vs Control: L2 Name	All Nodes	Stat	1.32E+04	1.19E+04	1.47E+04	1.39E+04	1.66E+04	1.33E+04	1.92E+04	1.66E+04	1.27E+04	1.47E+04
		pval	1.65E-06	1.52E-03	2.38E-12	1.99E-09	7.39E-23	8.21E-11	2.70E-42	7.39E-23	7.09E-06	3.44E-12
		Δmean	5.78E-01	-5.37E-04	2.94E-01	3.53E-01	4.48E+01	7.38E-01	6.69E+01	4.48E+01	1.34E+03	3.19E-01
	Singletons Removed	Stat	4.83E+03	1.26E+03	6.34E+03	5.54E+03	8.27E+03	4.96E+03	1.09E+04	8.27E+03	2.06E+03	6.31E+03
		pval	9.56E-02	0.239	9.56E-02	0.911	3.08E-09	0.000136	3.16E-31	3.08E-09	0.000274	0.112
		Δmean	-6.84E-02	-7.35E-03	3.19E-02	-1.29E-03	3.23E+01	-1.13E-01	4.52E+01	3.23E+01	2.26E+03	3.65E-02
Male: AD vs Control: Diagnosis Name	All Nodes	Stat	1.29E+05	1.07E+05	1.28E+05	1.23E+05	1.29E+05	1.29E+05	1.29E+05	1.29E+05	1.07E+05	1.23E+05
		pval	2.85E-40	6.02E-13	3.40E-38	1.97E-32	1.05E-39	1.36E-40	9.84E-41	1.05E-39	2.27E-13	8.08E-32
		Δmean	7.64E-01	5.47E-04	1.74E-01	2.65E-01	4.58E+00	1.14E+00	3.15E+01	4.58E+00	2.61E+02	1.57E-01
	Singletons Removed	Stat	1.15E+03	1.00E+01	2.07E+02	5.52E+02	8.94E+02	1.13E+03	1.36E+03	8.94E+02	2.36E+02	2.91E+02
		pval	9.02E-04	2.24E-03	9.02E-04	1.70E-01	1.32E-01	1.93E-06	1.82E-06	1.32E-01	3.76E-03	1.98E-01
		Δmean	4.77E-01	-1.85E-01	-1.61E-01	1.26E-01	8.91E+00	7.43E-01	7.83E+01	8.91E+00	1.84E+03	-1.20E-01
Male: AD vs Control: L3 Name	All Nodes	Stat	1.29E+05	1.07E+05	1.28E+05	1.23E+05	1.29E+05	1.29E+05	1.29E+05	1.29E+05	1.07E+05	1.23E+05
		pval	2.85E-40	6.02E-13	3.40E-38	1.97E-32	1.05E-39	1.36E-40	9.84E-41	1.05E-39	2.27E-13	8.08E-32
		Δmean	7.64E-01	5.47E-04	1.74E-01	2.65E-01	4.58E+00	1.14E+00	3.15E+01	4.58E+00	2.61E+02	1.57E-01
	Singletons Removed	Stat	1.15E+03	1.00E+01	2.07E+02	5.52E+02	8.94E+02	1.13E+03	1.36E+03	8.94E+02	2.36E+02	2.91E+02
		pval	0.000902	0.00224	9.02E-04	1.70E-01	1.32E-01	1.93E-06	1.82E-06	1.32E-01	3.76E-03	1.98E-01
		Δmean	4.77E-01	-1.85E-01	-1.61E-01	1.26E-01	8.91E+00	7.43E-01	7.83E+01	8.91E+00	1.84E+03	-1.20E-01
Male: AD vs Control: L2 Name	All Nodes	Stat	1.30E+04	1.17E+04	1.40E+04	1.35E+04	1.58E+04	1.28E+04	1.85E+04	1.58E+04	1.24E+04	1.41E+04
		pval	6.41E-07	8.24E-04	8.73E-11	4.08E-09	4.30E-20	8.55E-10	5.46E-40	4.30E-20	4.95E-06	3.24E-11
		Δmean	5.91E-01	-3.78E-04	2.75E-01	3.55E-01	3.95E+01	7.12E-01	6.31E+01	3.95E+01	1.18E+03	3.08E-01
	Singleton Removed	Stat	5.00E+03	1.23E+03	6.01E+03	5.37E+03	7.81E+03	4.82E+03	1.05E+04	7.81E+03	1.96E+03	5.97E+03
		pval	2.61E-01	0.0909	2.61E-01	0.872	2.80E-07	5.81E-05	5.09E-29	2.80E-07	0.00993	0.232
		Δmean	-4.56E-02	-7.47E-03	2.05E-02	2.06E-03	2.75E+01	-1.26E-01	4.20E+01	2.75E+01	1.80E+03	2.81E-02
Male vs Female Controls: Diagnosis Name	All Nodes	Stat	9.73E+04	9.74E+04	9.73E+04	9.74E+04	9.73E+04	9.73E+04	9.73E+04	9.73E+04	9.74E+04	9.74E+04
		pval	0.863	0.903	0.863	0.881	0.863	0.863	0.863	0.863	0.903	0.881
		Δmean	-0.00251	-0.000158	-0.00101	-0.00087	-0.00507	-0.0037	-0.00716	-0.00507	-0.00974	-0.000757
	Singletons Removed	Stat	3.20E+01	7.50E+00	3.20E+01	18	3.20E+01	3.20E+01	3.20E+01	3.20E+01	8.00E+00	18
		pval	0.957	1	0.957	0.933	0.957	0.95	0.958	0.957	0.876	0.935
		Δmean	0	-2.78E-17	0	0	0	0	0	0	0	0
Male vs Female Controls: L3 Name	All Nodes	Stat	5.71E+04	5.80E+04	5.73E+04	5.75E+04	5.74E+04	5.59E+04	5.76E+04	5.74E+04	5.80E+04	5.77E+04
		pval	0.661	0.958	0.731	0.789	0.76	0.297	0.824	0.76	0.973	0.84
		Δmean	-0.0298	-0.00017	-0.00736	-0.00852	-0.00692	-0.0989	-0.551	-0.00692	3.69	-0.00383
	Singletons Removed	Stat	7.10E+03	7.34E+02	7.30E+03	5.43E+03	7.39E+03	5.82E+03	7.57E+03	7.39E+03	7.60E+02	5.57E+03
		pval	0.847	0.799	0.847	0.946	0.727	4.94E-07	0.492	0.727	0.996	0.802
		Δmean	-0.00638	-0.00132	0.00195	-0.00083	0.686	-0.193	1.6	0.686	40.6	0.00488
Male vs Female Controls: L2 Name	All Nodes	Stat	9.63E+03	9.77E+03	9.80E+03	9.81E+03	9.80E+03	9.68E+03	9.82E+03	9.80E+03	9.77E+03	9.91E+03
		pval	0.798	0.955	0.994	0.984	0.998	0.84	0.98	0.998	0.958	0.868
		Δmean	-0.0198	-0.000146	-0.00259	-9.77E-05	0.205	-0.0245	-0.274	0.205	-0.956	0.00301
	Singleton Removed	Stat	3.19E+03	8.50E+02	3.37E+03	3.26E+03	3.36E+03	3.24E+03	3.38E+03	3.36E+03	8.52E+02	3.36E+03
		pval	0.775	0.93	0.775	0.933	0.794	0.801	0.744	0.794	0.915	0.687
		Δmean	-0.0114	-0.000286	0.00475	0.00146	0.815	-0.0123	0.323	0.815	10	0.00653

Color Legend

- pval < .05
- pval < .01
- pval < .001
- pval < .0001
- positive Δmean
- negative Δmean

Supplementary Table 3. All Diagnosis Network Metrics

An attached excel sheet with 3 tabs. **Tab 1:** Diagnosis Networks are created with nodes representing a diagnostic category or diagnosis shared among >1% of patients in a group, and edges representing >1% of co-diagnosis in a group. **Tab 2 (UCSF) and 3 (Mount Sinai):** Network metrics are computed for nodes in each network, and the distribution of metrics are compared between networks. Comparisons are performed with and without the removal of singletons (single nodes with no neighbors). A Mann-Whitney U-test is performed to compare the distribution of each network metric, with colors based upon p-value cutoff. The mean difference in metric between comparison groups is also shown.

Supplementary Figures

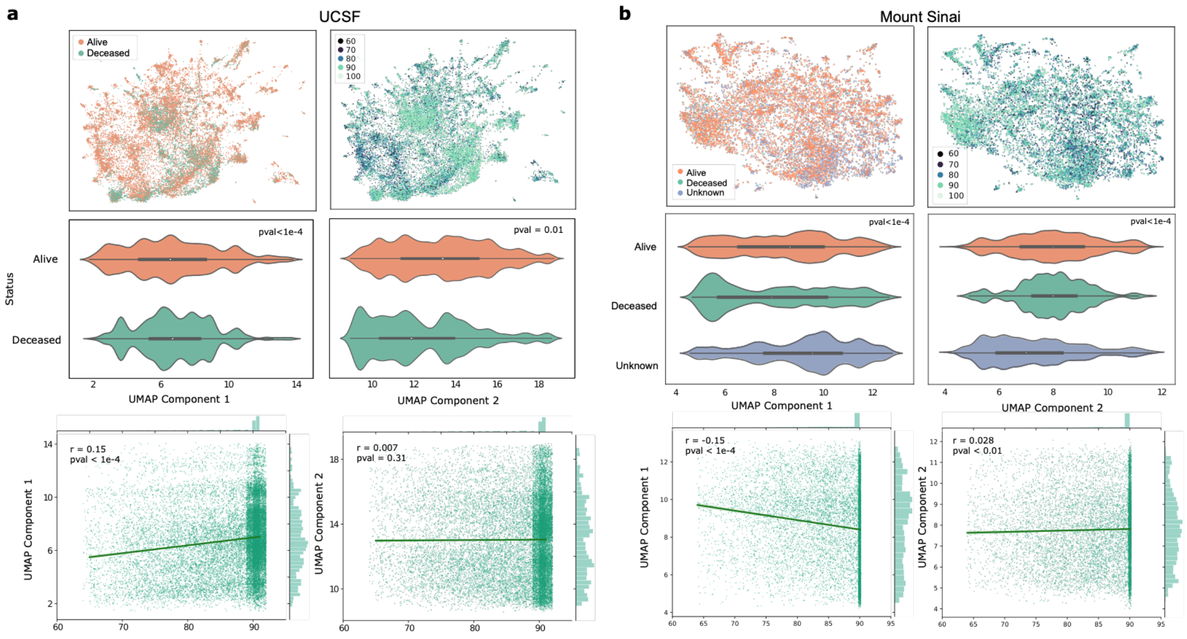

Supplementary Figure 1. Demographic correlation across UMAP principal components.

a. The top two graphs show the UMAP of AD and control cohorts at UCSF, colored by deceased status (left) and estimated age (right). The middle graphs show distribution of deceased status among the two UMAP components, which are compared with a two-sided Mann-Whitney U-Test. The bottom graphs show estimated age across the two UMAP components, with marginal distributions shown on the sides. A regression line is plotted, and a Pearson's R correlation test is performed.

b. The same UMAP plots are shown as in **a**, but for Mount Sinai.

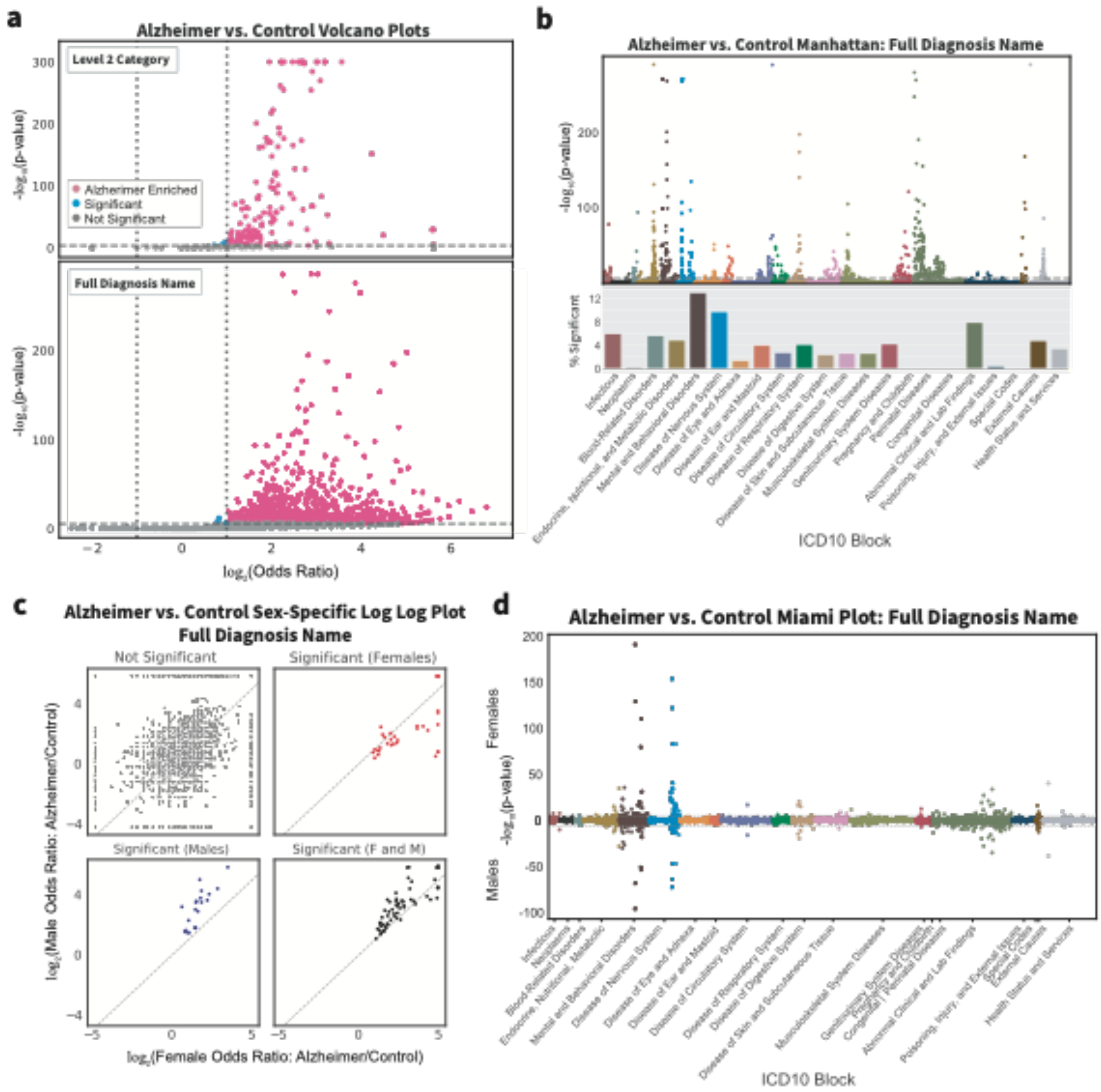

Supplementary Figure 2. Comorbidity Enrichment Analysis identifies enriched diagnosis in Patients with AD vs. Controls and Sex-Specific Enrichments at Mount Sinai.

a. Volcano plot for Level 2 categories (top) and full diagnosis names (bottom) compared between AD and control cohorts using two-sided Fisher Exact or Chi-Squared test. P-value cutoff is Bonferroni corrected at 0.05 with log2 odds ratio cutoff at 1 for AD enriched (pink)

and remaining significant diagnoses in blue.

b. Above, a Manhattan plot with full diagnosis names colored by ICD-10-CM categories with significance determined by two-sided Fisher Exact or Chi Square Test with Bonferroni-corrected p-value cutoff of 0.05. Bottom, percentage of diagnosis in each ICD-10-CM category that is significant.
c. Full diagnosis names compared between patients with AD and controls within each sex. The log of the odds ratio is plotted on the axis, and points are colored by significance (two-sided Fisher Exact or Chi Square test with Bonferroni-corrected p-value cutoff of 0.05).
d. Miami plot of the diagnosis names grouped by sex and ICD-10-CM categories.

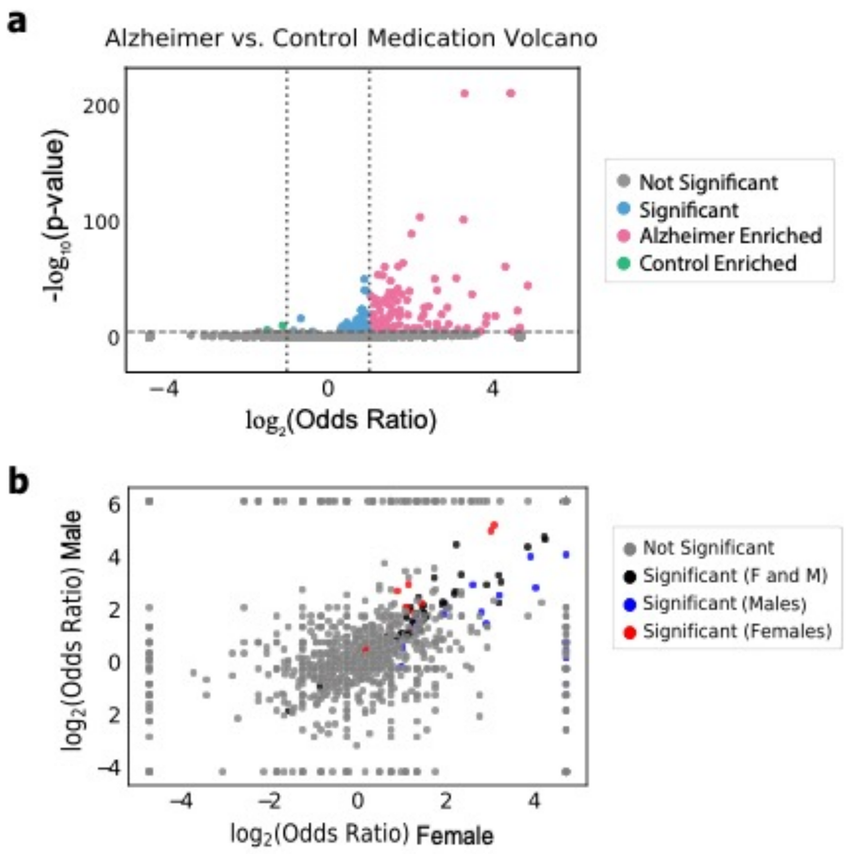

Supplementary Figure 3. Medication Enrichment Analysis identifies Enriched Medications between AD and Control Cohorts.

a. Volcano plot for generic medication names compared between patients with AD and controls using two-sided Fisher Exact or Chi-Squared Test. P-value cutoff is Bonferroni-corrected at 0.05 with odds ratio cutoff at 2 for AD enriched (pink) or 1/2 for controlled enriched (green). Remaining significant diagnoses are in blue.

b. Log-log plot of generic medication names compared between patients with AD and controls within each sex. The log of the odds ratio for each sex is plotted on the axis, with points colored by significance (Bonferroni-corrected p-value of 0.05) if female only (red), male only (blue), or both (black).

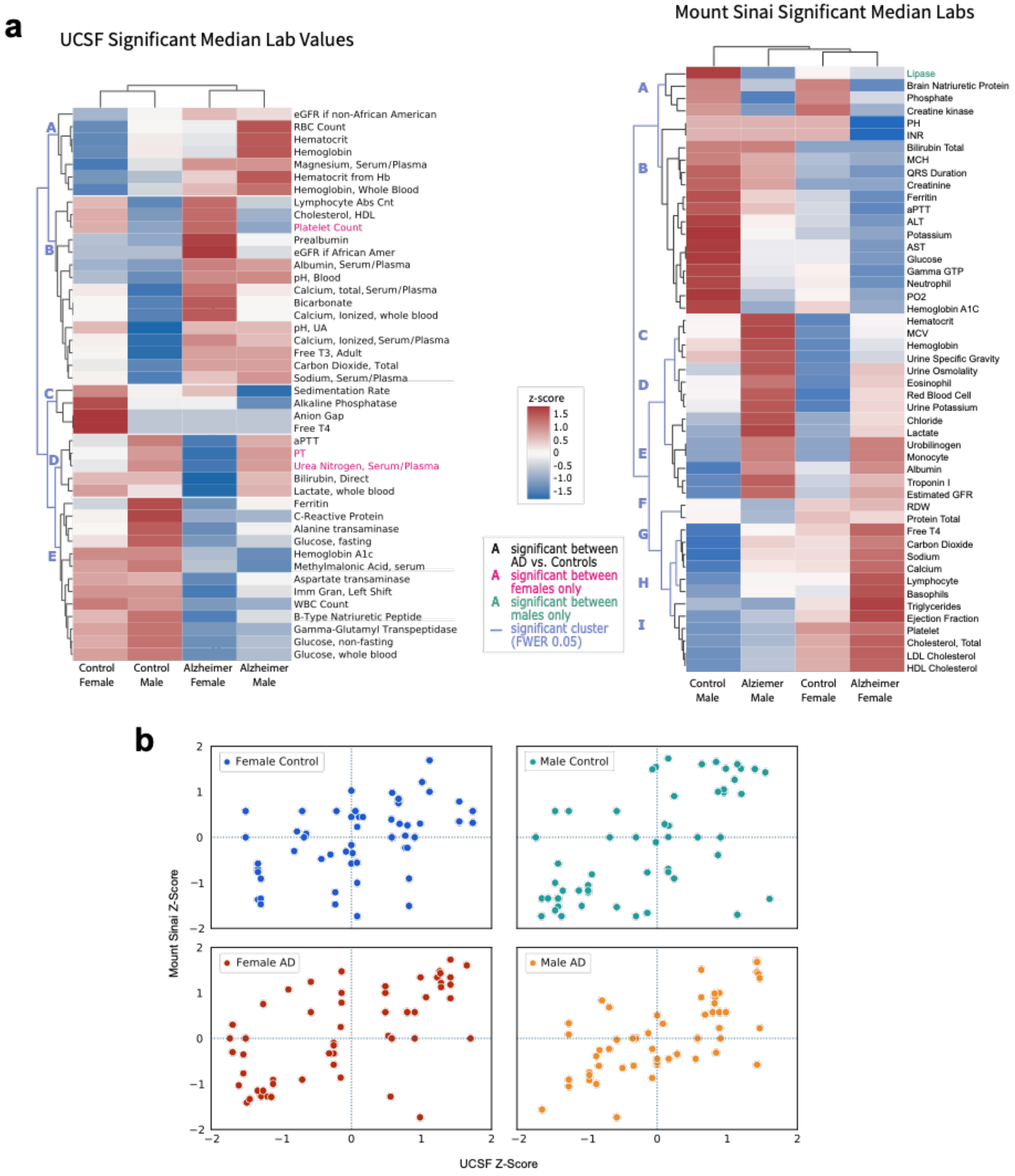

Supplementary Figure 4. Stratifying by AD status and sex allows identification of lab trends between groups.

a. Heatmap of lab values filtered on significance at UCSF in AD vs control comparison across

sex-specific groups. Labs are clustered with light blue lines representing significant cluster breaks (FWER corrected p-value 0.05). Text color represents significant labs among females only (pink), or significant between patients with AD vs control patients (black). Heatmap colors represent z-score of the average median value across the 4 groups.

b. Heatmap of lab values filtered on significance at Mount Sinai in AD vs control comparison across sex-specific groups. Labs are clustered with light blue lines representing significant cluster breaks (FWER corrected p-value 0.05). Text color represents significant labs among males only (green), or significant between patients with AD vs control patients (black). Heatmap colors represent z-score of the average median value across the 4 groups.
c. Comparison of z-scored lab values between UCSF and Mount Sinai showing significant correlations within each AD/sex-stratified groups. Female control: Spearman $\rho = 0.45$, p-value < 0.001; Male control: 0.46, p-value < 0.001; Females with AD: 0.59, p-value < 1e-5; Males with AD: 0.64, p-value < 1e-5.
