# Supplementary material for: Deep phenotyping of Alzheimer’s disease leveraging electronic medical records identifies sex-specific clinical associations
Source: Nat Commun. 2022 Feb 3;13:675. doi: 10.1038/s41467-022-28273-0 (PMC8814236; doi:10.1038/s41467-022-28273-0)
Supplement: Supplementary file 2 — Reporting Summary [file 41467_2022_28273_MOESM2_ESM.pdf]

nature research

Corresponding author(s): Alice Tang, Marina Sirota

Last updated by author(s): Dec 8, 2021

Reporting Summary

Nature Research wishes to improve the reproducibility of the work that we publish. This form provides structure for consistency and transparency in reporting. For further information on Nature Research policies, see our Editorial Policies and the Editorial Policy Checklist.

Statistics

For all statistical analyses, confirm that the following items are present in the figure legend, table legend, main text, or Methods section.

n/a	Confirmed	
☐	☒	The exact sample size (n) for each experimental group/condition, given as a discrete number and unit of measurement
☒	☐	A statement on whether measurements were taken from distinct samples or whether the same sample was measured repeatedly
☐	☒	The statistical test(s) used AND whether they are one- or two-sided<br>*Only common tests should be described solely by name; describe more complex techniques in the Methods section.*
☒	☐	A description of all covariates tested
☐	☒	A description of any assumptions or corrections, such as tests of normality and adjustment for multiple comparisons
☐	☒	A full description of the statistical parameters including central tendency (e.g. means) or other basic estimates (e.g. regression coefficient) AND variation (e.g. standard deviation) or associated estimates of uncertainty (e.g. confidence intervals)
☐	☒	For null hypothesis testing, the test statistic (e.g. F, t, r) with confidence intervals, effect sizes, degrees of freedom and P value noted<br>*Give P values as exact values whenever suitable.*
☒	☐	For Bayesian analysis, information on the choice of priors and Markov chain Monte Carlo settings
☐	☒	For hierarchical and complex designs, identification of the appropriate level for tests and full reporting of outcomes
☐	☒	Estimates of effect sizes (e.g. Cohen's d, Pearson's r), indicating how they were calculated

Our web collection on statistics for biologists contains articles on many of the points above.

Software and code

Policy information about availability of computer code

Data collection	No new data was collected for this study. No software was used for data collection.
Data analysis	See Methods

For manuscripts utilizing custom algorithms or software that are central to the research but not yet described in published literature, software must be made available to editors and reviewers. We strongly encourage code deposition in a community repository (e.g. GitHub). See the Nature Research guidelines for submitting code & software for further information.

Data

Policy information about availability of data

All manuscripts must include a data availability statement. This statement should provide the following information, where applicable:

- Accession codes, unique identifiers, or web links for publicly available datasets
- A list of figures that have associated raw data
- A description of any restrictions on data availability

Data was taken from the de-identified UCSF electronic medical records database and the Mount Sinai electronic medical records database.
Raw clinical datasets are not available due to patient privacy concerns. Aggregated data can be accessed in the supplements or visualized in the Rshiny app https://vizad.org.

Field-specific reporting

Please select the one below that is the best fit for your research. If you are not sure, read the appropriate sections before making your selection.

☒ Life sciences ☐ Behavioural & social sciences ☐ Ecological, evolutionary & environmental sciences

For a reference copy of the document with all sections, see nature.com/documents/nr-reporting-summary-flat.pdf

Life sciences study design

All studies must disclose on these points even when the disclosure is negative.

Sample size	No sample size calculation was performed prior to study. Sample size was determined by the number of patients with Alzheimer's Disease in the UCSF (8804) and Mount Sinai (5958) EMR database. A 1 to 2 matching ratio was utilized to identify controls in order to ensure sufficient power to capture common comorbidities in the population.
Data exclusions	No data was excluded from analysis, but our identified cohort included patients over 64 years of age in order to capture late onset AD.
Replication	Not applicable, this study did not acquire new data. Replication of analyses were performed in the Mount Sinai EMR.
Randomization	Not applicable, this study did not acquire new data.
Blinding	Not applicable, this study did not acquire new data.

Reporting for specific materials, systems and methods

We require information from authors about some types of materials, experimental systems and methods used in many studies. Here, indicate whether each material, system or method listed is relevant to your study. If you are not sure if a list item applies to your research, read the appropriate section before selecting a response.

Materials & experimental systems

n/a	Involved in the study	
☒	☐	Antibodies
☒	☐	Eukaryotic cell lines
☒	☐	Palaeontology and archaeology
☒	☐	Animals and other organisms
☐	☒	Human research participants
☒	☐	Clinical data
☒	☐	Dual use research of concern

Methods

n/a	Involved in the study	
☒	☐	ChIP-seq
☒	☐	Flow cytometry
☒	☐	MRI-based neuroimaging

Human research participants

Policy information about studies involving human research participants

Population characteristics	Study participants were identified from the UCSF and Mount Sinai EMR, which includes patients seen at both institutions. Cohorts include patients over 64 years of age. Propensity score matched control participants were also identified from the UCSF and Mount Sinai EMR databases. Demographic characteristics are shown in Table 1.
Recruitment	No recruitment was performed in this study.
Ethics oversight	Analysis of UCSF data was performed by employees of UCSF under Institutional Review Board approval (IRB - 20-32422). All data was de-identified. Analysis of Mount Sinai data was performed by employees of the Icahn School of Medicine at Mount Sinai under Institutional Review Board approval (IRB-19-02369). All data was de-identified and was obtained from the Mount Sinai Data Warehouse. Since all clinical data was de-identified, written informed consent was waived by the institutions.

Note that full information on the approval of the study protocol must also be provided in the manuscript.
