# Supplementary material for: Deep phenotyping of Alzheimer’s disease leveraging electronic medical records identifies sex-specific clinical associations
Source: Nat Commun. 2022 Feb 3;13:675. doi: 10.1038/s41467-022-28273-0 (PMC8814236; doi:10.1038/s41467-022-28273-0)
Supplement: Supplementary file 3 — Description of Additional Supplementary Files [file 41467_2022_28273_MOESM3_ESM.pdf]

Title: Supplementary Data 1.
Description: Thresholded Full Tables of Diagnosis Enrichment Analysis.

An excel sheet with 3 levels of diagnosis categories and sex-specific analysis (6 tabs) for each institution (12 tabs total). Lists include diagnosis enriched between AD and control cohorts, and sex-specific enrichments. Diagnoses are thresholded to represent > 10 patients, with un-corrected p-values (from two-sided Fisher Exact or Chi Square test) and odds ratios. The data from UCSF can be visualized and explored in the Rshiny app: vizad.org.

Title: Supplementary Data 2.
Description: Encounter Controlled Diagnosis Enrichment Analysis.

An excel sheets with 3 levels of diagnosis categories on encounter-controlled control cohorts (described in Methods) and sex-specific analysis at UCSF (6 tabs). Lists include diagnoses enriched between AD and control cohorts, and sex-specific enrichments. Diagnoses are thresholded to represent > 10 patients, with un-corrected p-values (from two-sided Fisher Exact or Chi Square test) and odds-ratios. The data can be visualized and explored in the Rshiny app: vizad.org.
